# Insights into the Associations Between Systolic Left Ventricular Rotational Mechanics and Left Atrial Peak Reservoir Strains in Healthy Adults from the MAGYAR-Healthy Study

**DOI:** 10.3390/biomedicines12112515

**Published:** 2024-11-04

**Authors:** Attila Nemes, Árpád Kormányos, Nóra Ambrus, Csaba Lengyel

**Affiliations:** Department of Medicine, Albert Szent-Györgyi Medical School, University of Szeged, Semmelweis Street 8, P.O. Box 427, H-6725 Szeged, Hungary; kormanyos.arpad@med.u-szeged.hu (Á.K.); ambrusnora@gmail.com (N.A.); lecs@in1st.szote.u-szeged.hu (C.L.)

**Keywords:** left ventricular, rotation, left atrial, strain, three-dimensional, echocardiography

## Abstract

Introduction: In systole, when the left ventricle (LV) twists, the left atrium (LA) behaves like a reservoir, having a special wall contractility pattern opposite to that of the LV wall. Accordingly, the objective of the present study was to investigate the associations between LV rotational mechanics and LA peak (reservoir) strains as assessed simultaneously by three-dimensional speckle-tracking echocardiography (3DSTE) under healthy conditions. Methods: In the present study, 157 healthy adults (mean age: 33.2 ± 12.7 years, 73 men) were involved. Complete two-dimensional Doppler echocardiography with 3DSTE-derived data acquisition were performed in all cases. The 3DSTE-derived LV rotational and LA strain parameters were determined at a later date. Results: Global LA peak reservoir circumferential (22.7 ± 6.4% vs. 27.6 ± 6.8%, *p* < 0.05) and area (57.8 ± 20.0% vs. 66.0 ± 22.7%, *p* < 0.05) strains proved to be reduced in the case of the highest vs. lowest basal LV rotation; other LA peak reservoir strains were not associated with increasing basal LV rotation. Global LA peak radial strain was highest in the case of the lowest vs. highest apical LV rotation (−19.2 ± 9.4% vs. −13.0 ± 8.2%, *p* < 0.05). Global LA peak reservoir 3D strain was lowest in the case of the highest vs. lowest apical LV rotation (−9.9 ± 6.8% vs. −5.0 ± 4.2%, *p* < 0.05). Only apical LV rotation proved to be significantly reduced in the case of the highest vs. lowest global LA peak reservoir 3D strain (8.12 ± 3.23° vs. 10.50 ± 3.44°, *p* < 0.05). Other global LA peak reservoir strains were not associated with basal and apical LV rotations. Conclusions: In LV systole, LV rotational mechanics is associated with LA deformation represented by LA peak (reservoir) strains even in healthy circumstances. While basal LV rotation is associated with LA widening, apical LV rotation is associated with LA thinning, suggesting the close cooperation of the LV and LA in systole even in healthy adults.

## 1. Introduction

It is well known that the heart cycle consists of two parts: diastole and systole. In the latter, the left ventricle (LV) is emptied through the aortic valve with the mitral valve closed [1]. The movement of the LV is complex during systole, as in addition to wall contractions represented by deformation parameters [2], it twists like the wringing of a towel at the same time as well. The apical part of the LV rotates in the counterclockwise direction, and its base rotates in the clockwise direction. With this form of movement, the rate of ejection is optimized [3,4,5,6,7]. During LV twist or rotational mechanics, the left atrium (LA) behaves like a reservoir, and its volume is the largest. In this phase of LA function, the direction of its wall movement is opposite to that of the LV wall, and it can be quantitatively characterized using strains. In systole, thinning, widening and lengthening of LA walls in the radial, circumferential and longitudinal directions can be detected, and these changes are represented by specific so-called LA peak or reservoir strains, respectively [2,8,9]. The fact is that systolic LV rotational mechanics and simultaneous LA contractility, and the deformation parameters representing them, have not been sufficiently investigated within clinical circumstances. Three-dimensional (3D) speckle-tracking echocardiography (3DSTE) provides an easy-to-learn/easy-to-perform method with a non-invasive approach to examining the above mechanisms and creates an opportunity to perform physiological studies at the same time [10,11,12,13]. Accordingly, the objective of the present study was to examine the association between 3DSTE-derived characteristics of LV rotational mechanics and LA peak reservoir strains under healthy conditions by examining what would happen when these parameters were smaller or greater than the average as well. Whether the regional LV rotations showed a correlation with different deformation parameters representing the 3D contractility of the LA in systole was also investigated.

## 2. Subjects and Methods

### 2.1. Subject Population

In this study, 157 healthy adult individuals (mean age: 33.2 ± 12.7 years, 73 men) were involved, with LV rotational mechanics being in the normal direction being confirmed in all individuals. The enrollment of all volunteers was carried out between 2011 and 2017. In all cases, laboratory testing, physical examination, standard 12-lead electrocardiography (ECG) and two-dimensional (2D) Doppler echocardiography were performed with negative results. No one used any medications or was a smoker, professional athlete or obese. If someone had a disease or clinical condition that could have affected the results, they were excluded from the study. The 3DSTE-based data acquisition was conducted at the same time as 2D Doppler echocardiography in accordance with the latest guidelines and practices. The detailed 3DSTE-based analysis was carried out offline at a later date [1,2,3,4]. The present retrospective study is part of the ‘Motion Analysis of the heart and Great vessels bY three-dimensionAl speckle-tRacking echocardiography in Healthy subjects’ study (MAGYAR-Healthy Study), which was organized partly to perform physiological analyses between 3DSTE-derived parameters in healthy adults (‘Magyar’ means ‘Hungarian’ in the Hungarian language). This study was conducted in accordance with the Declaration of Helsinki (as revised in 2013). All participants gave informed consent and the Institutional and Regional Human Biomedical Research Committee of the University of Szeged, Hungary (No.: 71/2011), approved this study.

### 2.2. Two-Dimensional Doppler Echocardiography

The Toshiba Artida^TM^ echocardiography tool was used on all individuals (Toshiba Medical Systems, Tokyo, Japan), attached to a PST-30BT (1–5 MHz) phased-array transducer for a complete analysis. Complete routine 2D echocardiographic assessment of heart chambers and valves was conducted in all healthy individuals. Studies included the quantification of the LA and LV, exclusion of significant valve stenoses and regurgitations by Doppler echocardiography, and pulsed Doppler assessment of early (E) and late (A) diastolic mitral inflow velocities and their ratios (E/A) [1]. 

### 2.3. Three-Dimensional Speckle-Tracking Echocardiography

The same Toshiba Artida^TM^ cardiac ultrasound device attached to a PST-25SX matrix transducer with 3D capability was used for the 3DSTE scans. During the studies, subjects in sinus rhythm lay in the left lateral decubitus position, and then 3D echocardiographic datasets were acquired from an apical window, while the subject held his/her breath. To achieve optimal images, 6 subvolumes were acquired over 6 cardiac cycles, and the software automatically combined these subvolumes into a full-volume 3D echocardiographic dataset. Detailed data analysis was carried out offline at a later time using version 2.7 of the manufacturer’s 3D Wall Motion Tracking software (Ultra Extend, Toshiba Medical Systems, Tokyo, Japan) [10,11,12,13].

### 2.4. Determination of LV Rotational Mechanics

**Figure 1:**
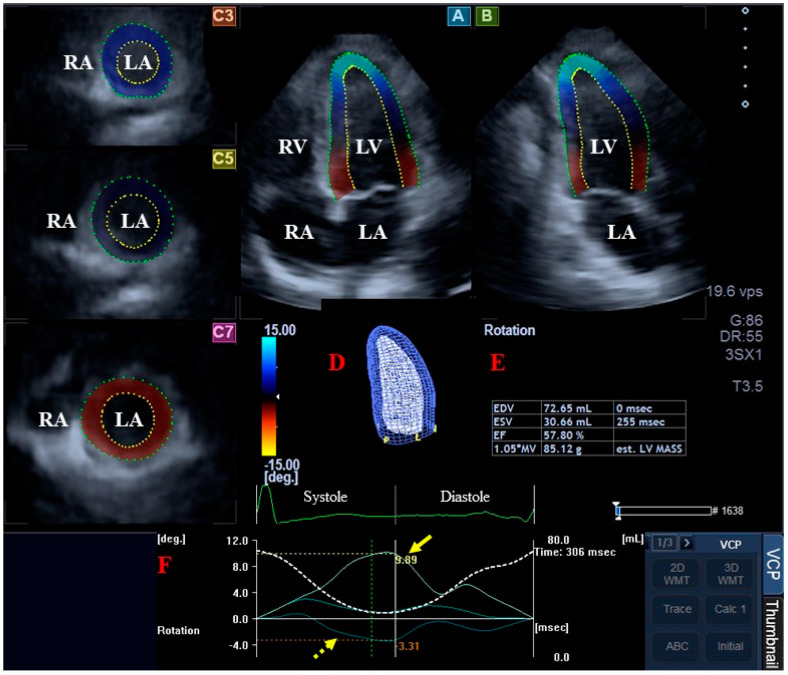
Three-dimensional (3D) speckle-tracking echocardiography-derived evaluation of the left ventricle (LV): apical four-chamber (**A**) and apical two-chamber long-axis views (**B**), and short-axis view at basal (**C3**), midventricular (**C5**) and apical LV levels (**C7**) are demonstrated together with a 3D cast of the LV (**D**) and calculated LV volumetric data (**E**). Apical (yellow arrow) and basal LV rotational curves (dashed yellow arrow) together with LV volume change curve during the heart cycle (dashed white curve) (**F**). Abbreviations. EDV, end-diastolic volume; EF, ejection fraction; ESV, end-systolic volume; LA, left atrium; LV, left ventricle; RA, right atrium; RV, right ventricle.

To quantify the LV rotational mechanics, apical longitudinal views were selected in apical 4-chamber (AP4CH) and 2-chamber (AP2CH) views, as well as basal, midventricular and apical cross-sectional planes, and the mitral annular (MA) ring—LV edges and endocardial surface of the LV apex were determined. Then, after sequential analysis, a virtual 3D cast of the LV was created (Figure 1) [14]. According to our own practice, we defined the following features of LV rotational mechanics from the same 3D cast of the LV:

- clockwise basal LV rotation (in degrees);
- counterclockwise apical LV rotation (in degrees);
- LV twist (net difference in LV apical and basal rotations in degrees);
- time-to-peak LV twist (in milliseconds).

If the direction of the LV apical and basal rotations were the same, clockwise or counterclockwise, LV twist was absent. This phenomenon is called LV ‘rigid body rotation’ (RBR). Individuals with LV-RBR were excluded from the present study [14].

### 2.5. Determination of LA Strains

By using the same software, at the end of the diastole, AP4CH and AP2CH views, as well as 3 short-axis views at the apical, midatrial and superior LA levels, were automatically selected on LA-focused images. After image optimizations, the endocardial LA boundary was detected by adjusting multiple markers from the edge of the septum-MA towards the edge of the LV lateral wall-MA. Then, automatic reconstruction of the LA endocardial surface was performed, allowing strain analyses. Several global LA strains representing the whole LA were determined [2,8,9]:

#### 2.5.1. Unidimensional/Unidirectional Global LA Strains

- Radial strain (GRS) representing thickening and thinning of the myocardial walls;
- Longitudinal strain (GLS) representing lengthening and shortening of the myocardial walls;
- Circumferential strain (GCS) representing widening and narrowing of the myocardial walls.

#### 2.5.2. Multidimensional/Multidirectional Complex LA Strains

- Area strain (GAS) is a combination of GLS and GCS.
- 3D strain (G3DS) is a combination of GRS, GLS and GCS.

**Figure 2:**
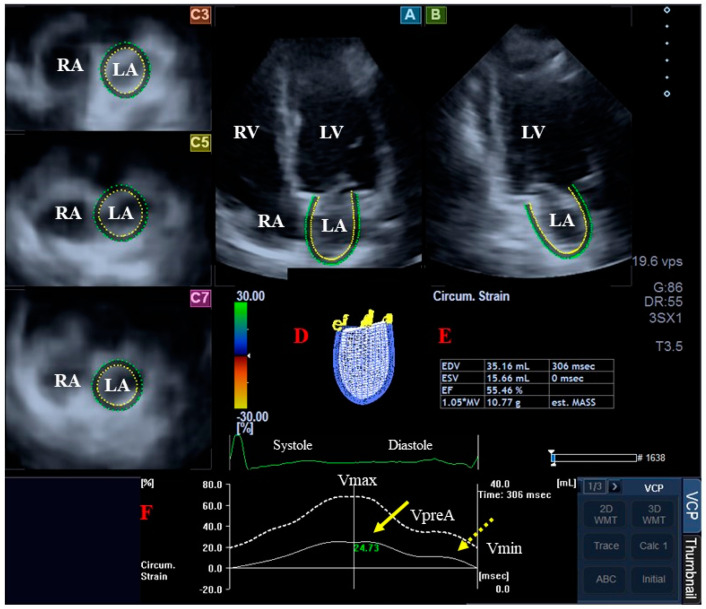
Three-dimensional (3D) speckle-tracking echocardiography-derived evaluation of the left atrium (LA): apical four-chamber (**A**) and two-chamber long-axis views (**B**), and short-axis view at basal (**C3**), midatrial (**C5**) and superior LA levels (**C7**) demonstrated with a 3D cast of the LA (**D**) and calculated LA volumetric data (**E**). Time—global LA volume change (dashed white curve) and time—LA global longitudinal strain change (white curve) respecting the heart cycle are demonstrated (**F**). LA peak reservoir strains and LA strains at atrial contraction are presented with yellow and dashed yellow arrows, respectively. Abbreviations. EDV, end-diastolic volume, EF, ejection fraction, ESV, end-systolic volume, LA, left atrium, LV, left ventricle, RA, right atrium, RV, right ventricle, Vmax, maximum volume of the LA, VpreA, preatrial contraction LA volume, Vmin, minimum volume of the LA.

Twin-peak strain curves were detected, where the first represented reservoir function of the LA in systole, while the second represented atrial contraction at end-diastole (LA systole) (Figure 2). In the present study, systolic LA peak reservoir strains were used for analysis [2,8,9].

### 2.6. Statistical Analysis

The mean ± standard deviation (SD) format was used for continuous variables, while the number/percent format was used for categorical variables. The difference was significant if *p* < 0.05. The normality was tested by the Shapiro–Wilk test: in the case of a normal distribution of continuous variables, Student’s *t*-test with Welch correction was used, while when distribution was non-normal, the Mann–Whitney–Wilcoxon test was performed. Intra- and inter-observer agreements were tested by interclass correlation coefficients (ICCs). SPSS software was used during statistical analyses (SPSS Inc., version 22, Chicago, IL, USA).

## 3. Results

### 3.1. Clinical and Two-Dimensional Doppler Echocardiographic Data

From the echo data, the LA diameter was measured in the parasternal long-axis view (36.8 ± 4.0 mm) together with LV end-diastolic diameter (48.1 ± 3.7 mm) and volume (106.5 ± 22.8 mL), LV end-systolic diameter (40.8 ± 26.2 mm) and volume (36.3 ± 9.0 mL), interventricular septum (8.9 ± 1.6 mm), LV posterior wall (9.1 ± 1.7 mm), and LV ejection fraction (66.0 ± 4.9%), all were being in the normal ranges. The mean E/A proved to be 1.37 ± 0.38. Significant valvular stenosis or valvular regurgitation larger than grade 1 could not be confirmed in any valves in any cases.

### 3.2. Classification of Subjects

3DSTE-derived LA peak reservoir strains and parameters featuring the LV rotational mechanics of healthy individuals are demonstrated in Table 1. The group of healthy controls were classified into three subgroups according to their normal basal and apical LV rotations, peak LA-GRS, LA-GCS, LA-GLS, LA-G3DS and LA-GAS: the estimated mean ± SD served as the lower (2.18 degree, 5.95 degree, 6.73%, 18.33%, 17.43%, 1.75% and 39.63%, respectively) and larger (6.34 degree, 13.03 degree, 22.73%, 49.31%, 35.51%, 13.07% and 96.41%, respectively) values. 

### 3.3. Increase in Basal or Apical LV Rotations

Increased basal LV rotation is associated with a decreasing tendency of rotation of the LV apex and increased LV twist. Increased rotation of the LV apex is associated with a decreasing tendency of rotation of the LV base and increased LV twist (Table 2). 

### 3.4. Increased LV Rotations and LA Peak Reservoir Strain

Peak LA-GCS and LA-GAS proved to be reduced in the case of the highest basal LV rotation, while other LA peak reservoir strains did not show an association with increasing basal LV rotation. Peak LA-GRS was highest in the case of the lowest apical LV rotation. Peak LA-3DS was lowest in the case of the highest apical LV rotation (Table 2).

### 3.5. Increase in LA Peak Reservoir Strains

All LA peak reservoir strains showed an increasing tendency of the other strains but to different extents. With increasing peak LA-GRS, increasing peak LA-GLS, LA-GCS and LA-GAS could be detected, but only up to a point, beyond which further increase could not be detected (except peak LA-G3DS). Increasing peak LA-GCS was associated with increase in all other strains except for peak LA-G3DS (only up to a point). An increase in all LA peak reservoir strains could be seen with increasing peak LA-GLS, except for peak LA-GRS (only up to a point). From complex strains, with increasing peak LA-G3DS, increasing peak LA-GLS, LA-GCS and LA-GAS could be detected, but only up to a point, beyond which further increases could not be detected (except peak LA-GRS). An increase in all LA peak reservoir strains could be detected with increasing peak LA-GAS, but only up to a point, beyond which further increases in LA peak reservoir strains could not be detected (Table 3 and Table 4).

### 3.6. Increased LA Peak Reservoir Strains and Basal and Apical LV Rotations

Only apical LV rotation proved to be significantly reduced in the case of the highest peak LA-G3DS. Other global LA peak reservoir strains did not show any associations with apical and basal rotations of the LV (Table 3 and Table 4).

### 3.7. Feasibility of 3DSTE-Derived Measurements

The frame rate proved to be 31 ± 2 fps. This study comprised 308 healthy individuals, but 151 cases were excluded due to the image quality being insufficient for LA and/or LV quantifications. According to this fact, the overall feasibility proved to be 51%.

### 3.8. Reproducibility of 3DSTE-Derived Assessments

The mean ± SD difference in rotations of the LV base and apex and LA peak reservoir strains as assessed by 3DSTE simultaneously were assessed two times by the same observer and by two independent observers together with ICCs (Table 5).

## 4. Discussion

In left ventricular (LV) systole, the coordinated complex movement of heart chambers and valves is observed. Due to the spatial structure of the LV, its systolic pump function is the result of the coordinated work of the muscle fibers that make up the LV. However, due to the oblique muscle LV fibers running perpendicular to each other, not only can contraction be detected in systole, but the basal region of the LV rotates in the clockwise direction and the apical region of the LV rotates in the counterclockwise direction, having a specific ‘towel-wringing’ like movement, a phenomenon that is called LV twist. This sort of motion seems to be responsible for approximately 40% of ejection [3,4,5,6,7]. While the cavity of the LV is the smallest in end-systole, its walls thicken, narrow and shorten in the radial, circumferential and longitudinal directions, represented by specific LV strains. In the left atrium (LA), wall segments move in the opposite direction, ensuring reservoir function in systole. The LA cavity is the largest in end-systole, explaining why this phase is called ‘reservoir’, while thinning, lengthening and widening of the LA walls in the radial, longitudinal and circumferential directions represented by negative LA global radial strain (GRS) and positive LA global circumferential strain (GCS) and LA global longitudinal strain (GLS) are seen, respectively [1,2,3,8,9]. Due to the fact that the interaction of certain LV and LA features has not been deeply examined, to the best of the authors’ knowledge, this is the first time where a three-dimensional (3D) speckle-tracking echocardiography (3DSTE)-derived analysis of the relationship between LV rotational mechanics and LA reservoir deformation represented by LA peak strains were performed in healthy adult volunteers to determine their relationship.

3DSTE seems to be an ideal method for the simultaneous analysis of LV rotational mechanics and LA deformation at the same time. 3DSTE is a non-invasive, non-radiating, easy-to-learn and easy-to-implement procedure, which can be performed several times within a relatively short time, even in healthy subjects. These advantages make it possible to perform physiological studies based on 3DSTE [10,11,12,13]. In the past, simultaneous routine 3D measurement of quantitative features of LV twist represented by apical and basal LV rotations and LA deformation represented by its strains could not be quantified, and only two-dimensional (2D)-projected parameters could be calculated in specific planes determined by the observer (1). This sort of approach of evaluation was based on certain predetermined assumptions, which could theoretically challenge the accuracy of the measurements (1). Therefore, according to recent recommendations, 2D speckle-tracking echocardiography (STE)-derived determination of LV rotational mechanics is not recommended, and only 3D echocardiography is suggested [10,11,12,13,14]. Although numerous studies have demonstrated the clinical possibility and importance of LA strain determination during 2DSTE, their 3DSTE-based determination seems to be closer to reality. In addition, 3DSTE is validated and normal reference values of LV rotational mechanics and LA strains measured in healthy subjects are also available [14,15,16,17,18,19].

In a recent study, strong associations between LA volumes and LV rotational mechanics were found [20]. The rotation of the LV base was highest in the presence of the highest maximum LA volume in end-systole (and the LA preatrial contraction volume in early diastole), and the rotation of the LV apex did not associate obviously with any LA volumes. End-systolic LA volume did not relate to LV rotations, but the highest end-systolic rotation of the LV base was associated with increased diastolic LA volumes, and reduced diastolic LA volumes were seen in the case of increased rotation of the LV apex. These findings could theoretically explain that blood flowing into the LV in diastole results in increased basal (not apical) LV rotation, as this region is closest to the LA outflow/LV inflow. End-systolic apical LV rotation was associated with lower volumes of the LA, suggesting that it may play a role in lower atrial preload [20].

With the presented findings, our knowledge was extended with several facts. The highest rotation of the LV base was associated with the lowest LA-GCS (LA widening) and LA-GAS. Moreover, with higher apical LV rotation, lower LA-GRS (LA thinning) and LA-G3DS were associated. With increasing LA peak reservoir strains, neither basal nor apical LV rotations showed associations except for the rotation of the LV apex, which was lowest in the presence of the highest LA-G3DS.

To determine the clinical importance of a phenomenon in a specific abnormality, better understanding in healthy circumstances has a significant importance. The presented results suggest a complex adaptation to changes in the volumes and functional properties of the LA and LV, suggesting that each left heart chamber can affect the other, even in healthy circumstances. It must also be noted that certain parameters (volumes, functional characteristics, etc.) are not independent of each other, so they must be examined together, which can make analysis very complicated. Therefore, further multi-parameter investigations are warranted in healthy subjects. Moreover, similar studies are also needed to examine these relationships in certain pathologies, which are associated with changes in LA and/or LV morphology and function. Future studies examining patients with early systolic LV abnormalities (e.g., cases with reduced LV-GLS but preserved LV ejection fraction) would be worthwhile to investigate which relationship/association disappears the earliest and which still exists in healthy subjects based on the present research. This sort of analysis could help us form a deeper understanding of the development of the early stages of heart failure.

During this study’s analyses, many factors were not taken account in order to highlight the most important ones, e.g., the roles of age, gender difference, heart rate and blood pressure were not investigated. Accordingly, an even more complex approach would be necessary to understand the presented physiological associations.

## 5. Limitations

The limitations presented as follows were the most important ones of this study. 

Firstly, the sample size could have been larger to allow analyses like the evaluation of gender differences or subjects being in different age decades.

According to a consensus document, measurement of global LA strain is recommended and radial (or transverse) strain is not recommended. However, due to the experimental nature of this analysis, a full analysis was performed [21].

Image quality was an important aspect. Under current technical conditions, 2D echocardiography still has a significant advantage, which limits the usability of 3DSTE. In the present study, the overall feasibility proved to be only 51%. Further improvements are needed to improve the temporal and spatial resolution of the images taken [10,11,12,13,22].

3DSTE is capable of evaluating LV strain parameters simultaneously using the same 3D echocardiography dataset. A detailed analysis of LV/LA strains would have exceeded the limits of the present paper and could well be the subject of another study.

This study did not purpose to assess associations between LA volumes and LV rotational mechanics due to the detailed investigation of this topic [20].

The validation of calculated parameters was not among the objectives of the current study due to their validated nature [15,16,17,18].

The pulmonary veins and the LA appendage were excluded from the evaluations. 

Morphological abnormalities of the LA were not aimed to be characterized.

3DSTE-derived evaluation of the volumes and functional properties of other heart chambers were not to be performed either.

In this study, analyses were performed with the atrial septum as part of the LA.

Finally, both parameters of LV rotational mechanics and LA strains had specific age and gender dependencies, which could have affected the findings [14,19].

## 6. Conclusions

In LV systole, LV rotational mechanics is associated with LA deformation, represented by LA peak reservoir strains even in healthy circumstances. While basal LV rotation is associated with LA widening, apical LV rotation is associated with LA thinning, suggesting the close cooperation of the LV and LA in systole even in healthy adults.

## Figures and Tables

**Table 1:** Three-dimensional speckle-tracking echocardiography-derived left atrial peak reservoir strains and left ventricular rotational parameters.

Parameter	Measure
Left Atrial Volumes	
Maximum Left Atrial Volume (mL)	40.5 ± 12.8
Preatrial Contraction Left Atrial Volume (mL)	27.4 ± 11.3
Minimum Left Atrial Volume (mL)	19.1 ± 7.8
Systolic Left Atrial Peak Reservoir Strains	
Left Atrial Global Radial Strain (%)	−14.7 ± 8.0
Left Atrial Global Circumferential Strain (%)	33.8 ± 15.5
Left Atrial Global Longitudinal Strain (%)	26.5 ± 9.0
Left Atrial Global Three-Dimensional Strain (%)	−7.3 ± 5.7
Left Atrial Global Area Strain (%)	68.0 ± 28.4
Left Ventricular Volume-Based Parameters	
End-Diastolic Left Ventricular Volume (mL)	85.6 ± 23.2
Body Surface Area-Indexed End-Diastolic Left Ventricular Volume (mL/m^2^)	46.5 ± 13.1
End-Systolic Left Ventricular Volume (mL)	36.1 ± 10.5
Body Surface Area-Indexed End-Systolic Left Ventricular Volume (mL/m^2^)	19.6 ± 5.6
Left Ventricular Ejection Fraction (mL)	58.3 ± 5.7
Left Ventricular Mass (g)	158.1 ± 32.0
Systolic Left Ventricular Rotational Mechanics	
Basal Left Ventricular Rotation (Basal LVrot_,_ °)	−4.26 ± 2.08
Apical Left Ventricular Rotation (Apical LVrot_,_ °)	9.49 ± 3.54
Left Ventricular Twist (LVtwist_,_ °)	13.75 ± 3.70
Time-to-LVtwist (ms)	344 ± 130

**Table 2:** Peak left atrial strains and left ventricular rotational parameters in different basal and apical left ventricular rotations groups.

	Basal LV Rotation ≤ −2.18° (n = 17)	−2.18° < Basal LV Rotation < −6.34° (n = 116)	−6.34° ≤ Basal LV Rotation (n = 23)	Apical LV Rotation ≤ 5.95° (n = 24)	5.95° < Apical LV Rotation < 13.03° (n = 106)	13.03° < Apical LV Rotation (n = 27)
LA-GRS (%)	−13.3 ± 7.2	−14.5 ± 7.6	−16.8 ± 9.9	−19.2 ± 9.4	−14.2 ± 7.2 ^‡^	−13.0 ± 8.2 ^‡^
LA-GLS (%)	30.7 ± 13.7	35.1 ± 16.3	30.0 ± 11.8	35.2 ± 14.4	34.0 ± 15.3	31.9 ± 17.0
LA-GCS (%)	27.6 ± 6.8	27.1 ± 9.6	22.7 ± 6.4 *^,†^	24.0 ± 7.1	27.3 ± 9.4	25.2 ± 8.5
LA-G3DS (%)	−7.0 ± 5.1	−7.1 ± 5.4	−9.0 ± 6.9	−9.9 ± 6.8	−7.5 ± 5.5	−5.0 ± 4.2 ^‡,#^
LA-GAS (%)	66.0 ± 22.7	70.5 ± 20.2	57.8 ± 20.0 ^†^	66.6 ± 23.5	69.2 ± 28.2	64.7 ± 32.6
Basal LV rotation (°)	−1.52 ± 0.50	−3.84 ± 1.07 *	−8.09 ± 1.16 *^,†^	−5.05 ± 2.10	−4.25 ± 2.08	−3.60 ± 1.76 ^‡^
Apical LV rotation (°)	10.93 ± 3.45	9.51 ± 3.50	8.37 ± 3.50 *	4.58 ± 0.97	9.11 ± 1.76 ^‡^	15.37 ± 1.90 ^‡,#^
LV twist (°)	12.5 ± 3.5	13.35 ± 3.44	16.45 ± 3.73 *^,†^	9.63 ± 2.27	13.36 ± 2.57 ^‡^	18.98 ± 2.49 ^‡,#^
Time-to-LV twist (ms)	276 ± 92	355 ± 140 *	339 ± 84 *	318 ± 150	349 ± 131	345 ± 106

* *p* < 0.05 vs. basal LV rotation ≤ 2.18 degree; ^†^ *p* < 0.05 vs. 2.18 degree < basal LV rotation < 6.34 degree; ^‡^ *p* < 0.05 vs. apical LV rotation ≤ 5.95 degree; ^#^ *p* < 0.05 vs. 5.95 degree < apical LV rotation < 13.03 degree. Abbreviations. LA, left atrial; LV, left ventricular; G3DS, global three-dimensional strain; GAS, global area strain; GCS, global circumferential strain; GLS, global longitudinal strain; GRS, global radial strain.

**Table 3:** Left atrial peak reservoir strains and left ventricular rotational parameters in different left atrial peak reservoir unidimensional strain groups.

	LA-GRS ≤ −6.73% (n = 22)	−6.73% < LA-GRS < −22.73% (n = 117)	−22.73% ≤ LA-GRS (n = 18)	LA-GCS ≤ 18.33% (n = 24)	18.33% < LA-GCS < 49.31% (n = 106)	49.31% ≤ LA-GCS (n = 27)	LA-GLS ≤ 17.43% (n = 23)	17.43% < LA-GLS < 35.51% (n = 110)	35.51% ≤ LA-GLS (n = 24)
LA-GRS (%)	−2.9 ± 2.3	−14.8 ± 4.6 *	−28.9 ± 6.6 *^,†^	−10.1 ± 7.0	−14.8 ± 7.8 ^‡^	−18.4 ± 7.6 ^‡,#^	−10.4 ± 8.6	−15.5 ± 8.1 ^$^	−15.4 ± 5.4 ^$^
LA-GLS (%)	24.2 ± 10.8	34.9 ± 15.4 *	38.2 ± 16.5 *	13.5 ± 3.6	31.9 ± 7.9 ^‡^	59.4 ± 10.5 ^‡,#^	22.2 ± 12.2	34.5 ± 15.2 ^$^	41.9 ± 12.9 ^$,&^
LA-GCS (%)	20.0 ± 8.4	27.7 ± 8.8 *	26.1 ± 8.1 *	18.1 ± 5.4	26.9 ± 8.2 ^‡^	32.2 ± 9.6 ^‡,#^	13.5 ± 3.4	26.0 ± 4.9 ^$^	41.3 ± 6.0 ^$,&^
LA-G3DS (%)	−0.8 ± 1.1	−7.6 ± 4.3 *	−14.1 ± 7.8 *^,†^	−4.6 ± 4.0	−7.9 ± 6.1 ^‡^	−7.9 ± 4.1 ^‡^	−4.9 ± 5.6	−7.5 ± 5.8 ^$^	−9.2 ± 4.4 ^$^
LA-GAS (%)	48.9 ± 25.5	71.2 ± 27.0 *	70.4 ± 31.2 *	31.7 ± 8.4	65.6 ± 18.2 ^‡^	110.0 ± 19.8 ^‡,#^	35.6 ± 17.2	68.2 ± 23.9 ^$^	98.3 ± 21.6 ^$,&^
Basal LV rotation (°)	−4.29 ± 1.66	−4.18 ± 2.09	−4.75 ± 2.37	−4.09 ± 1.87	−4.33 ± 2.18	−4.13 ± 1.77	−4.32 ± 2.14	−4.32 ± 2.15	−3.94 ± 1.56
Apical LV rotation (°)	9.54 ± 2.92	9.56 ± 3.64	9.00 ± 3.51	10.46 ± 3.78	9.18 ± 3.43	9.86 ± 3.55	9.67 ± 4.26	9.47 ± 3.48	9.45 ± 3.01
LV twist (°)	13.83 ± 3.00	13.74 ± 3.72	13.75 ± 4.28	14.55 ± 3.46	13.52 ± 3.72	13.99 ± 3.68	13.99 ± 4.04	13.78 ± 3.77	13.39 ± 2.86
Time-to-LV twist (ms)	339 ± 109	340 ± 116	374 ± 210	346 ± 91	351 ± 144	312 ± 95	342 ± 116	338 ± 127	368 ± 151

* *p* < 0.05 vs. LA-GRS ≤ −6.73%; ^†^ *p* < 0.05 vs. −6.73% < LA-GRS < −22.73%; ^‡^ *p* < 0.05 vs. LA-GCS ≤ 18.33%; ^#^ *p* < 0.05 vs. 18.33% < LA-GCS < 49.31%; ^$^ *p* < 0.05 vs. LA-GLS ≤ 17.43%; ^&^ *p* < 0.05 vs. 17.43% < LA-GLS < 35.51%. Abbreviations. LA, left atrial; LV, left ventricular; G3DS, global three-dimensional strain; GAS, global area strain; GCS, global circumferential strain; GLS, global longitudinal strain; GRS, global radial strain.

**Table 4:** Left atrial peak reservoir strains and left ventricular rotational parameters in different left atrial peak reservoir complex strain groups.

	LA-G3DS ≤ 1.75% (n = 27)	1.75% < LA-G3DS < 13.07% (n = 107)	13.07% ≤ LA-G3DS (n = 23)	LA-GAS ≤ 39.63% (n = 27)	39.63% < LA-GAS < 96.41% (n = 106)	96.41% ≤ LA-GAS (n = 24)
LA-GRS (%)	−6.8 ± 7.4	−14.8 ± 5.5 *	−24.0 ± 8.6 *^,†^	−10.4 ± 7.7	−15.2 ± 7.9 ^‡^	−17.6 ± 6.8 ^‡^
LA-GLS (%)	26.7 ± 12.8	35.6 ± 15.9 *	34.0 ± 13.8 *	15.9 ± 7.6	33.0 ± 9.1 ^‡^	57.7 ± 14.3 ^‡^
LA-GCS (%)	20.2 ± 7.9	28.2 ± 8.7 *	25.7 ± 8.6 *	16.0 ± 5.5	26.7 ± 6.2 ^‡^	37.3 ± 9.5 ^‡^
LA-G3DS (%)	−0.4 ± 0.5	−7.1 ± 3.1 *	−17.2 ± 4.6 *^,†^	−4.6 ± 4.8	−7.9 ± 5.9 ^‡^	−8.6 ± 4.6 ^‡^
LA-GAS (%)	49.7 ± 23.5	72.4 ± 28.1 *	69.0 ± 26.3	29.9 ± 7.5	66.8 ± 15.1 ^‡^	116.5 ± 15.2 ^‡^
basal LV rotation (°)	−4.23 ± 1.78	4.16 ± 2.03	−4.77 ± 2.50	−4.36 ± 1.92	−4.28 ± 2.20	−4.07 ± 1.58
apical LV rotation (°)	10.50 ± 3.44	9.53 ± 3.54	8.12 ± 3.23 *	10.1 ± 3.9	9.35 ± 3.48	9.45 ± 3.28
LV twist (°)	14.73 ± 3.72	13.70 ± 3.65	12.88 ± 3.64	14.5 ± 3.7	13.63 ± 3.65	13.52 ± 3.78
time-to-LV twist (ms)	327 ± 105	342 ± 123	369 ± 176	358 ± 104	346 ± 144	319 ± 75

* *p* < 0.05 vs. LA-G3DS ≤ 1.75%; ^†^ *p* < 0.05 vs. 1.75% < LA-G3DS < 13.07%; ^‡^ *p* < 0.05 vs. LA-GAS ≤ 39.63%. Abbreviations. LA, left atrial; LV, left ventricular; G3DS, global three-dimensional strain; GAS, global area strain; GCS, global circumferential strain; GLS, global longitudinal strain; GRS, global radial strain.

**Table 5:** Intra- and inter-observer agreement for three-dimensional speckle-tracking echocardiography-derived apical and basal left ventricular rotations and left atrial peak reservoir strains in healthy adults.

	Intra-Observer Agreement		Inter-Observer Agreement	
	Mean ± 2SD Difference in Values Obtained by Two Measurements of the Same Observer	ICC Between Measurements of the Same Observer	Mean ± 2SD Difference in Values Obtained by 2 Measurements of the Same Observer	ICC Between Measurements of the Same Observer
Apical LV Rotation (°)	0.6 ± 0.4	0.81 (*p* < 0.001)	0.7 ± 0.7	0.80 (*p* < 0.001)
Basal LV Rotation (°)	0.3 ± 0.2	0.82 (*p* < 0.001)	0.3 ± 0.3	0.81 (*p* < 0.001)
LA-GRS (%)	−1.9 ± 11.1	0.86 (*p* < 0.001)	−4.0 ± 10.4	0.83 (*p* < 0.001)
LA-GLS (%)	4.7 ± 15.8	0.82 (*p* < 0.001)	4.9 ± 16.1	0.80 (*p* < 0.001)
LA-GCS (%)	3.1 ± 7.5	0.81 (*p* < 0.001)	3.6 ± 8.9	0.79 (*p* < 0.001)
LA-GAS (%)	6.7 ± 34.7	0.81 (*p* < 0.001)	11.1 ± 35.3	0.79 (*p* < 0.001)
LA-G3DS (%)	−1.3 ± 10.2	0.82 (*p* < 0.001)	−2.1 ± 9.4	0.80 (*p* < 0.001)

Abbreviations. LA = left atrial, LV = left ventricular, G3DS = global three-dimensional strain. GAS = global area strain, GCS = global circumferential strain, GLS = global longitudinal strain, GRS = global radial strain, ICC = interclass correlation coefficient.

## Data Availability

The manuscript has not been published elsewhere. Data are contained within the article.
